# Contribution of Extensive Farming Practices to the Supply of Floral Resources for Pollinators

**DOI:** 10.3390/insects11110818

**Published:** 2020-11-20

**Authors:** Alban Langlois, Anne-Laure Jacquemart, Julien Piqueray

**Affiliations:** 1 Earth and Life Institute, Agronomy Université Catholique de Louvain, Croix-du-Sud 2, Box L7.05.14, 1348 Louvain-la-Neuve, Belgium; anne-laure.jacquemart@uclouvain.be; 2 Natagriwal Asbl, Passage des Déportés 2, 5030 Gembloux, Belgium; jpiqueray@natagriwal.be

**Keywords:** agri-environment schemes, extensive agriculture, floral resources, landscape scale, nectar, pollinator conservation, bees

## Abstract

**Simple Summary:**

One of the causes of pollinator decline is the decreased availability of flower resources, that constitute their nutritional requirements. In particular, the intensification of agricultural practices has led to a loss of flower resources. For many years, as part of the Common Agricultural Policy and the efforts to preserve biodiversity, several Agri-Environmental Schemes (AESs) and extensive farming practices have been promoted in Europe. To assess the relative contribution of extensive farming practices such as hedgerows, organic crops and extensive grasslands, we compared pairs of agricultural landscapes in Belgium. We recorded the densities of the insect-pollinated plant species per biotope and per month, the abundance and diversity of the main visiting insects. In April, hedgerows and forest edges constituted the main nectar resources. In May, most of the nectar resources were produced by grasslands and mass-flowering crops. In June, extensive grasslands and organic crops contributed to nectar resources, contrarily to intensive agricultural elements. Extensive and diverse agricultural practices should therefore be encouraged to provide less fluctuating nectar resources on a landscape scale.

**Abstract:**

Intensification of agricultural practices leads to a loss of floral resources and drives pollinator decline. Extensive agricultural practices are encouraged in Europe and contribute to the preservation of biodiversity. We compared three agricultural landscapes without extensive farming practices with three adjacent landscapes containing organic crops and extensively managed grasslands in Belgium. Nectar resource availability and plant–pollinator interactions were monitored from April to June. Flower density per plant species and plant–pollinator interactions were recorded in different landscape elements. In April, the main nectar resources were provided by linear elements such as hedgerows and forest edges. Nectar production peaked in May, driven by intensive grasslands and mass-flowering crops. Occurrence of extensive grasslands and organic crops significantly alleviated the nectar resource gap observed in June. Our results underscore the importance of maintaining landscape heterogeneity for continuous flower resources and highlight the specific role of extensive grasslands and organic crops in June.

## 1. Introduction

Since the beginning of the 20th century, agricultural yields have increased due to the intensification of agricultural practices, but this growth has come at the expense of biodiversity [1,2]. Among other groups, pollinators are declining in abundance and diversity worldwide [3]. Ecosystems rely on biodiversity for the delivery, stability, and resilience of ecosystem functions [4]. More than 80% of wild plant species and about 70% of crop species are totally or partially dependent on insects for pollination [5,6,7]. Therefore, pollinator decline has a dramatic effect on ecosystem functions, agricultural production, and food security [8,9,10]. The economic impacts of this decline are expected to be substantial. Pollination services have been valued at around 153 billion €/year worldwide and 22 billion €/year in Europe [11].

Destruction, fragmentation and degradation of habitats, climate changes, invasive species or pathogens, have been identified as the main drivers of pollinator decline [3,12,13]. The agricultural intensification has substantially altered the distribution, structure, and composition of habitats in agricultural landscapes. In croplands, fertilization and efficient weed control drastically alter the arable flora [14]. Intensification of grassland management has impoverished plant communities and resources for pollinators [15,16]. Due to land consolidation policies that include tree and hedgerow removal, small semi-natural elements such as hedgerows, ditches, hollow paths, and field margins have drastically decreased with the widespread of agricultural intensification since the early 20th century [17,18]. These modifications induce shifts or decreases in the quantity and quality of the floral resources available for pollinators in terms of the abundance of flowering plant species and their relative quantity and composition of nectar and pollen rewards [19,20,21]. Nectar and pollen provided by flowers are an important food source for pollinators such as bees who depends on nectar and pollen for growth, survival, reproduction, and resilience to stress [22,23]. Nectar consists mainly of sugars, whereas pollen is the main source of proteins and lipids [19,21,22,23].

Agriculture is therefore considered the largest contributor to biodiversity loss and pollinator decline [24,25,26]. The agricultural intensification has led to the simplification of the landscapes, including the rarefaction and degradation of semi-naturals habitats. Wild floral resources have become rarer and sparser in agricultural landscapes. Flower resources in agricultural landscapes are not homogeneously distributed in space and time [27]. Spatially, different vegetation elements provide valuable but variable resources, such as hedgerows and forest edges [28], extensively managed grasslands [29], arable weeds [30], road verges [31] or mass-flowering crops [32,33]. Pollinators like bees may have to forage further away from their nesting sites, thus increases the energetic cost of foraging that may impact insect fitness [34,35,36].

The amount and diversity of resources provided by these landscape elements vary in time according to the phenology of the different plant species [31,37,38]. Moreover, the seasonal variation in floral resources can reach critical levels that do not meet the needs of flower-feeding insects [22,37]. These food resources gaps may affect pollinator health and colony development, especially when combined with other stress factors [13,39,40].

As pollinators use different and complementary sources throughout the year to collect floral resources that meet their nutritional needs [31,41], both spatial and temporal availabilities of floral resources are, therefore, essential for pollinator survival.

To counteract biodiversity loss in agricultural landscapes, Agri-Environmental Schemes (AES) promoted by the EU Common Agricultural Policy (CAP) [42] have been implemented in the European Union. These schemes include measures such as sown wildflower strips that are specifically designed to preserve flower-feeding insects [21,43,44]. Other measures, such as organic farming and the extensive management of grasslands, could have indirect benefits for pollinators [45,46,47,48]. By promoting abundance and diversity of insect-pollinated plants and by limiting pesticide exposure, these practices are believed to be more favorable to pollinators than their intensive counterparts [29,30,47]. However, few studies have investigated the relative importance of AES and other extensive practices for floral resource supply at the agricultural landscape scale.

We do not know how these elements complement one another over the growing season at the landscape scale [31,37,41]. To set up effective measures for pollinators in agricultural landscapes, a better understanding of the spatio-temporal dynamics of floral resources is crucial [31,43,49,50,51,52,53,54]. Attempting to link landscape-scale nectar availability in time and space to pollinator activity should contribute importantly to our understanding of landscape-level management of various habitats for pollinator protection. This study aims to assess (i) the availability and distribution of floral resources in six contrasted agricultural landscapes; (ii) the links between floral resources and visitors; and (iii) the effect of extensive agricultural practices on floral resources and pollinators at the landscape scale.

## 2. Materials and Methods

### 2.1. Study Fields

The study was conducted in South Belgium, in three locations (Ychippe, Houyet and Wellin). The region has a temperate climate with a mean annual temperature of 9 °C and a mean annual precipitation around 900–1000 mm, evenly distributed along the year. The landscapes of this region are marked by alternating low hills (up to 300 m asl) and valleys. Land use is mainly agricultural and includes grasslands (25–35%) and croplands (10–35%) interspersed with villages and small towns. Forests cover around 30–40% of the land use.

**Figure 1:**
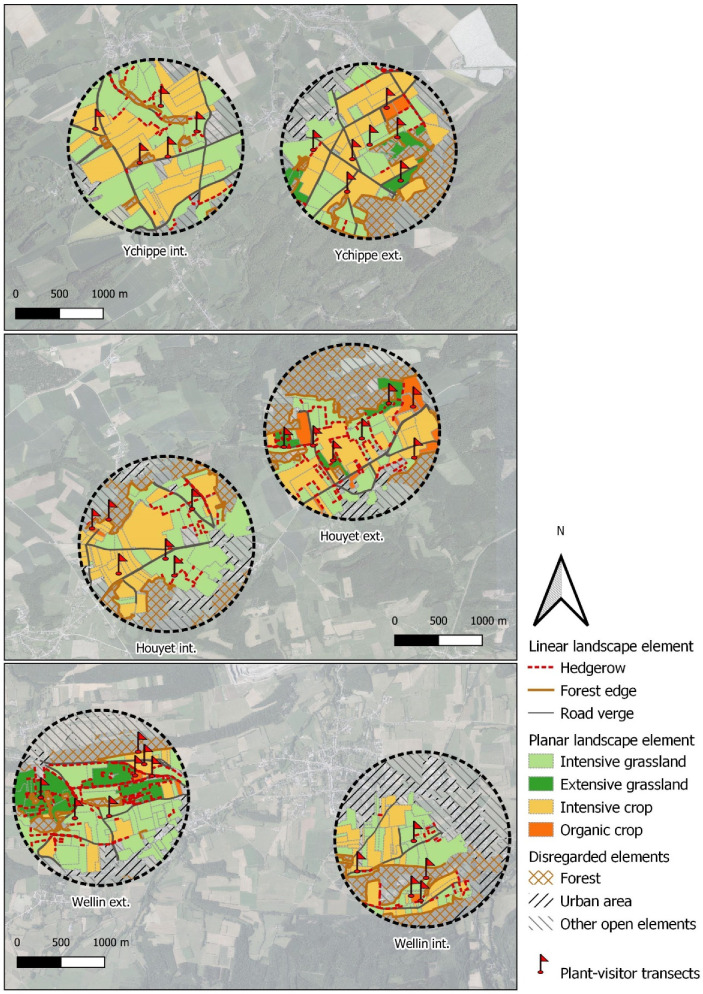
Location of the six studied agricultural landscapes. Hatched areas include not surveyed urbanized areas (including gardens and orchards), forests and other open elements. Locations of plant-visitor observation transects are indicated by red flags.

In order to estimate the floral resources available to visiting insects, six agricultural landscapes with a radius of 1 km each were considered (Figure 1). Insect foraging distance usually does not exceed 1 km [55], and we chose this scale as in similar studies [27,50,56]. Landscape locations represent local contrasts in Belgian agricultural management. We considered management to be ‘intensive’ when there was a low (<10%) proportion of (i) organic crops and (ii) extensive grasslands, i.e., semi-natural grasslands under Agri-Environmental Scheme (AES) with late mowing [57] or under Natura 2000 status with no fertilization, and no mowing or grazing before mid-June. We considered management to be ‘extensive’ when these practices were present at a higher (>10%) proportion. The six landscapes were chosen to create three pairs of contrasted landscapes (intensive vs. extensive) at a low geographical distance. Distances between landscapes of a pair varied from 2.4 km to 3.6 km (center to center). Within landscapes, we studied all agricultural biotopes.

Landscapes were subdivided into seven different landscape elements that were easily distinguishable: hedgerows, forest edges, road verges, intensive conventional and organic crops, intensive and extensive grasslands. In opposition to the extensive grasslands, intensive grasslands include pastures and hay meadows that are not subject to limitations on mowing, soil fertilization or livestock density. Due to restricted access, urbanized areas and forests were not considered in our surveys. The proportion of each landscape element was calculated using QGIS, version 3.4.12 (Table 1).

### 2.2. Floral Resources

This study focuses on nectar because it is the main energy source for pollinators, and provides a common unit (total sugar per flower) which can be used to denote the nutritional contribution of all plants [19,58].

Each month, from April to June 2019, flowering species cover and localization were assessed and mapped by walking through each landscape element and then digitalized using Qgis. Flowering species composition was therefore known for any landscape element patch in each study landscape, each month. We assessed the floral density for each flowering species, per month, for each landscape element type in which it grows. For each plant species in bloom on a given month, we randomly selected at least two populations (except if only one population was found at the landscape scale) per landscape in each type of element in which they were recorded. For instance, in May, *Lamium album* floral density was estimated in 17 road verges (from six landscapes), two hedgerows (from one landscape), three forest edges (from two landscapes, as in one of them only one population was found in forest edge). In order to have the most accurate estimation of the floral density, the size of the quadrat was adapted to the floral density of the species and its configuration in space. For planar elements, the quadrat size varied depending on the floral density in the element. When flower density was dense and homogeneous (>10 flowers/m²), we used 1 m² quadrats. When flower density was sparser (<10 flowers/m²), 25 m² (5 × 5 m) or 100 m² (10 × 10 m) quadrats were used.

For linear elements, a slightly different method was used to determine the number of flowers per square meter. For herbaceous species, the floral density was estimated using quadrats having the same area as for planar elements, but the shape was adapted to the linear configuration (i.e., 1 × 1 m, 1 × 25 m and 1 × 100 m quadrats). For trees or shrubs, flowers were counted in two 1 m^3^ volume randomly distributed on the tree crown at human height (1.70 m). The depth and height of the flowering parts of the plant (the crown for the trees and the whole plant for shrubs) were measured to obtain average dimensions for each tree or shrub species. The thickness of the flowering part was assumed to be 1 m for all species. For isolated individual plants (e.g., isolated *Prunus avium* in a *P. spinosa* hedgerow), the width of the plant was measured to determine the total volume of the flowering part. When there was more than one individual within 100 m of a linear element, the linear frequency (%) of the species over 100 m was estimated and the average height and depth of three random individuals were noted.

A total of 1596 density measurements were hence done for the whole study across the six study landscapes.

The flower abundance (number of flowers/floral unit) of each species at the landscape scale, each month, was extrapolated from density measurements and the flowering species mapping. We calculated the abundance of a given species at the landscape element patch scale by multiplying the average floral density of the species in the given landscape element type by the surface occupied by the patch. Values for all the landscape elements patches sheltering the species were then summed to provide the abundance of the species at the landscape level.

To evaluate nectar sugar production, flower abundance was multiplied by a single flower’s daily nectar sugar production. Values for the nectar sugar production of each species were taken from Baude et al. (2016) who measured or modelled the nectar sugar (sucrose) production per 24 h period per flower. Missing species were completed from Hick et al. (2016) and Ouvrard et al. (2018) [19,21,59].

Nectar sugar production was calculated at two scales: (i) nectar sugar productivity at the element level was the sum of the quantities of sugar produced by each flowering species present in a given element, divided by the total area occupied by that element. It is therefore the amount of nectar sugar produced by a virtual hectare of the element that would have an average species composition. (ii) The contribution of an element at the landscape scale was the average amount of nectar sugar produced in an element, weighted by the proportion of that element in a landscape.

### 2.3. Insect–Flower Interactions

We recorded the insect diversity foraging on each landscape element and the flowers they visited. From April to June 2019, we monthly recorded numbers of flower-visiting insects walking along one 100 m long transect per landscape element during 20 min. One 100 m transect was randomly placed in each landscape element at each site (Figure 1), for a total of 37 transects. The locations of these transects were maintained over the 3 months of the study.

To ensure that only foraging insects were counted, only insects visiting open flower units were recorded [21]. The insect observations were performed on the same day as the flower density estimations. At each sampling run, the order of transects during the day was randomized in order to avoid that a transect was always sampled at the same moment of the day. We focused on insects considered to be effective pollinators, i.e., *Hymenoptera, Lepidoptera, Diptera* and *Coleoptera*. When practicable, we identified insects to species level in the field (i.e., *Apis mellifera*, *Eristalis tenax*, etc.). Due to morphological similarities, we identified bumblebees in the field up to their Operational Taxonomic Unit (OTU). An OTU comprises a restricted group of species with the same type of coloration, see [60]. For analyses, pollinators were grouped into: syrphids, other dipterids, butterflies, other hymenopterans and non-corbiculate bees. Bumblebees have been grouped according to their OTU. It is likely that in some of the study sites, honeybee apiaries were located in private properties; this could not be verified.

### 2.4. Statistical Analyses

All analyses were conducted with R version 3.5.3 (R Core Development Team, 2018).

Nectar sugar production and pollinator abundance were compared between landscape elements using a mixed-effect model, with the studied landscape as a random factor. The effect of different landscape elements and months on the nectar production at the element level and pollinators abundance per transect were tested using General Linear Mixed Models (GLMM). The Poisson error distribution was applied to pollinators abundance data. Homoscedasticity and normality were improved by logarithmic transformation of nectar production data. Tukey’s post-hoc test for multiple comparisons of means was performed between landscape elements and nectar sugar production at the element level and pollinators abundances per transect, each month, separately. Post-hoc comparisons were conducted using Tukey contrasts within the GLMM models. Analyses were conducted using R packages ‘lme4’ (Bates et al., 2018) and ‘multcomp’ (Hothorn et al., 2008).

We then aimed at assessing the relationship between the nectar sugar production (μg m^−2^ days^−1^) of each plant species and the number of insects visits they received per species. For each plant species, the number of flower-visiting insects was computed from the insect-flower interactions monitoring. Nectar sugar production was estimated from the landscape flower densities and phenology. For each landscape element, the mean production value by plant species and by month was computed. For plant species flowering during two consecutive months, we averaged the monthly production weighted by the flower density and the number of occurrences in transects. We then computed the overall nectar sugar production of each species as the mean of landscape element production, weighted by the number of transects in the given landscape element.

Species nectar sugar production was used to explain the number of flower-visiting insects through a simple linear regression model. Normality of residuals was improved by log-transforming both nectar production and the number of flower-visiting insects. Analyses were conducted separately for the main visiting insect groups: honeybees (*Apis mellifera*), bumblebees (*Bombus* OTU), non-corbiculate bees, and syrphids (*Diptera Syrphidae*). Other insect groups were not considered here as data was insufficient. Only plant species visited at least once were considered for each insect group. For each pollinator group, the most over-visited plant species was the species having the highest positive values of residuals.

## 3. Results

### 3.1. Nectar Sugar Production

Across all six studied landscapes, we documented 33 entomophilous flowering species in April, 72 in May, and 126 in June. Nectar sugar production at the element level as well as nectar sugar contribution at the landscape scale varied significantly among elements (*p* < 0.01) and among months (*p* < 0.05; see Table 2). The interaction between elements and months was significant for nectar sugar production at the element level and for the element contribution to the production at the landscape scale (*p* < 0.01).

**Figure 2:**
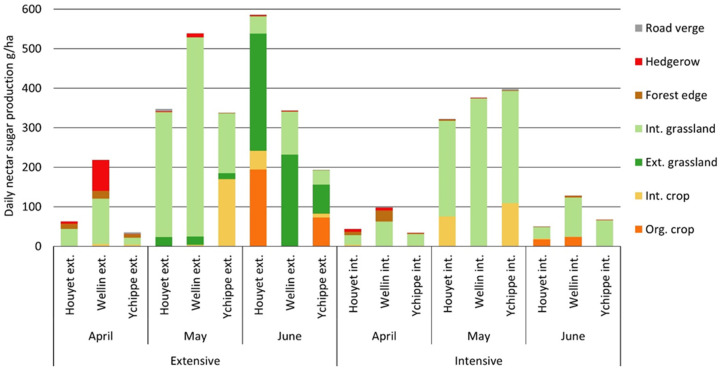
Daily nectar sugar production (g ha^−1^ day^−1^) at the landscape scale for each month. Each bar represents the sugar-production contribution of a given landscape element for one average hectare of the landscape. Ext., extensive, int., intensive, org., organic.

In April, at the landscape scale, mean daily nectar sugar production reached 82 ± 71 g ha^−1^ day^−1^. Average nectar sugar production was higher in extensive landscapes (105 ± 99 g ha^−1^ day^−1^) than in intensive landscapes (58 ± 34 g ha^−1^ day^−1^). Moreover, nectar sugar production was highly variable between extensive landscapes, from 35 to 218 g ha^−1^ day^−1^ (Figure 2).

In April, four species produced 92% of the total nectar sugar production, i.e., *Taraxacum* agg. (55%), *Salix* spp. (19%), *Prunus avium* (12%), and *P. spinosa* (7%), (Figure S1). *Taraxacum* agg. was mainly present in grasslands with a mean density of 10 capitula per square meter. Covering about 50 ± 6% of the total landscape surface (Table 1), grasslands provided 60% of the nectar sugar production (Table 2), followed by hedgerows (20%) and forest edges (16%). In these landscape elements, *Salix* spp., *P. avium*, and *P. spinosa* contributed to more than 99% of the nectar sugar production (Figure S1). On the contrary, intensive crops only produced 3% of the total nectar sugar production, mainly located in maize stubble. With an average nectar sugar production of 119 ± 76 g ha^−1^ day^−1^, the main nectar-producing species in maize stubble were *Lamium purpureum* (67%), *Veronica persica* (22%) and *Stellaria media* (8%).

In May, the average daily sugar production reached 387 ± 79 g ha^−1^ day^−1^. Total nectar sugar production did not differ between intensive and extensive landscapes (Table 3). Most of the nectar sugar (95%) was produced by two species, dandelions *Taraxacum* agg. in intensive grasslands (79%) and the mass flowering oilseed rape *Brassica napus* (17%) in intensive crops. With a nectar sugar production of 2367 g ha^−1^ day^−1^, *Brassica napus* was the best nectar sugar producer in the landscapes. Considering all crop species, intensive crop fields produced an average of 138 ± 156 g ha^−1^ day^−1^, including oilseed rape, cereals and maize stubble.

Road verges were the second most productive element in May as they contributed to a daily sugar production of 332 ± 300 g ha^−1^ day^−1^ (Table 2). However, due to their low area proportion at the landscape scale, they only accounted for 2% of the total nectar sugar production. The main nectar sugar producers were *Lamium album* (65%), *Taraxacum* agg. (10%), *Barbarea vulgaris* (8%), *Salix* spp. (7%), and *Anthriscus sylvestris* (5%).

In June, average daily nectar sugar production reached 228 ± 205 g ha^−1^ day^−1^. In extensive landscapes, nectar sugar production averaged 375 ± 198 g ha^−1^ day^−1^ whereas production reached only 82 ± 41 g ha^−1^ day^−1^ in intensive landscapes. In extensive landscapes, more than half of the nectar sugar production (53%) was produced by *Cirsium palustre* (mainly from extensive wet grasslands) and *Cyanus segetum* (from organic crop fields), (Figure S1). Extensive grasslands and organic crops occupied only an average of 20 % of the surface in extensive landscapes, and produced 77% of the nectar sugar production. In intensive landscapes, *Trifolium pratense* and *T. repens* were the main sources of nectar sugar and mainly occurred in intensive grasslands, which produced the majority (79%) of the nectar sugar in intensive landscapes.

### 3.2. Pollinator Observations

Throughout the study period, we recorded 1106 flower visitors, of which 282 involved Diptera (belonging to 11 families) and 796 involved Hymenoptera (11 families). Honeybees, *Apis mellifera* individuals were the most observed visitors (330 observations), followed by bumblebees *Bombus* spp. (278 individuals).

Among bumblebees, the main observed OTU were *Bombus terrestris* OTU (93 observations), *B. lapidarius* OTU (91 observations), and *B. pascuorum* OTU (81 observations). Due to limited time to insect observations, we recorded insect–flower interactions on only 72 of the 164 flowering entomophilous species. Ten of these species accounted for 66% of the insect–flower interactions. The most visited species were: *Rubus fruticosus* agg. (13%), *Lamium purpureum* (11%), *Cyanus segetum* (10%), *Taraxacum* agg. (7%), *Prunus spinosa* (7%), *Trifolium repens* (5%), *Heracleum sphondylium* (4%), *Veronica persica* (4%), *Brassica napus* (3%) and *Geranium pyrenaicum* (3%).

**Figure 3:**
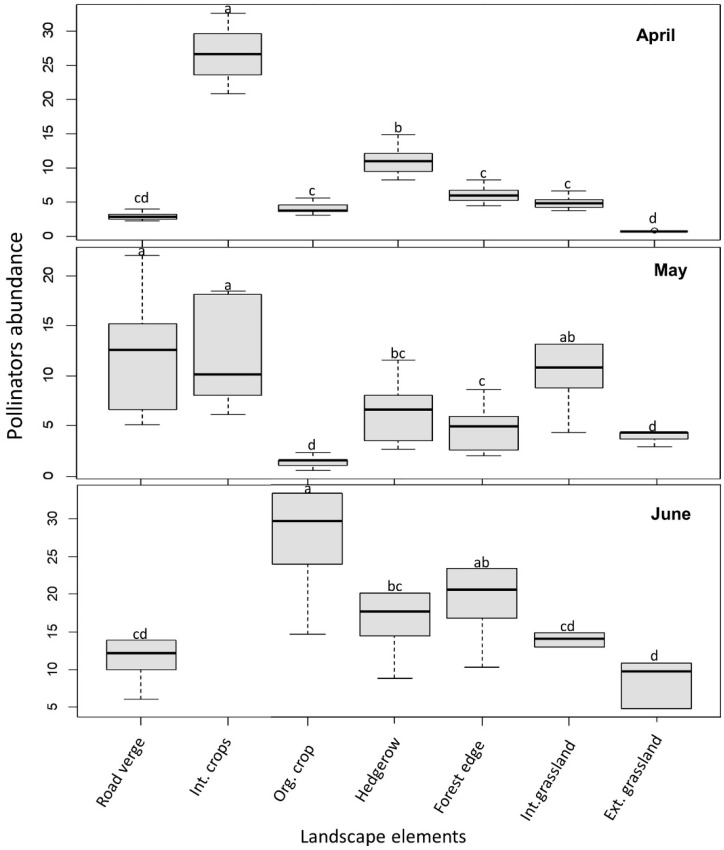
Pollinator abundance per transects across landscape elements. Different letters indicate significant differences among landscape elements. No insects were observed on intensive crops in June.

Pollinator abundance per landscape element varied significantly among elements (*N* = 84, *X*^2^ = 8.9, *p* = 0.0029), but also among months (*p* < 0.01; Figure 3). The interaction between landscape elements and months was significant for pollinator abundance (*p* < 0.01). However, pollinator abundance between intensive and extensive landscapes was not obviously different (Table 3).

**Figure 4:**
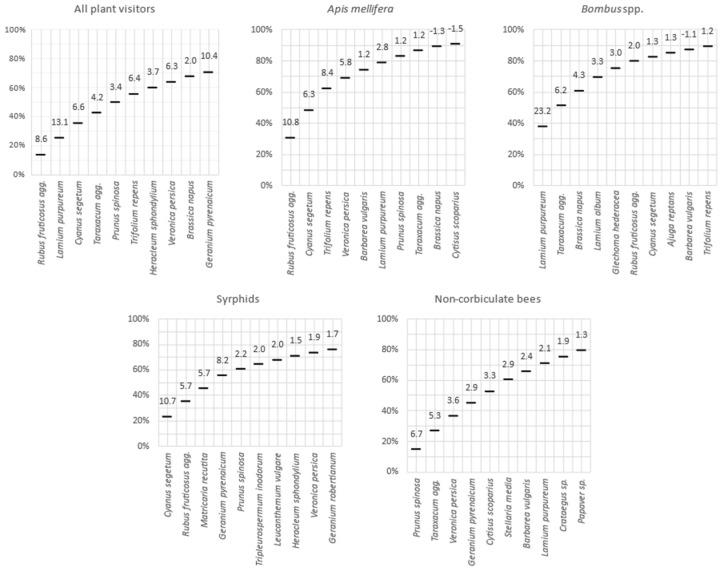
Cumulated number of visiting insects on 10 most visited plant species. Numbers above lines indicate the magnitude of the over-visitation (x times more visited than predicted by the simple linear regression model).

When considering all insect species together, we observed a significant positive relationship between nectar sugar production and number of flower-visiting insects per flower species (*p* < 0.001). This relationship was mainly due to *Apis mellifera*, which represents 30% of the observed individuals and for which the response to nectar sugar production was significant (*p* = 0.006). Conversely, when taken individually, other pollinator groups did not respond significantly to nectar sugar production *Bombus* spp. (*p* = 0.169), non-corbiculate bees (*p* = 0.585) and *Syrphids* (*p* = 0.223). In addition, the preference for particular flowering species differed between insect groups. *Apis mellifera* visited *Rubus fruticosus* agg. 11 times more often than predicted by nectar sugar production. Bumblebees were especially attracted to *Lamium purpureum* (23.2 times more than expected), while non-corbiculate bees visited *Prunus spinosa* 7 times more than expected and syrphids visited *Cyanus segetum* 11 times more than expected (Figure 4).

## 4. Discussion

### 4.1. Nectar Resource Variation in Space and Time

Our study showed strong seasonal trends in the distribution of floral nectar resources among landscape elements. In April, linear woody elements such as hedgerows and forest edges were the most productive landscape elements per unit area and produced significant resources at the landscape scale. *Prunus* spp. and *Salix* spp. were the main contributors to the nectar sugar production, as already shown in previous studies [37,61]. Later in the season, hedgerows and forest edges continuously produced nectar resources albeit to a lower extent than in early spring. Moreover, these elements provide nesting sites and are considered as buffer zones to protect pollinators from pesticide exposure [28,62]. Their reintegration into all agricultural landscapes is therefore to be strongly encouraged.

In May, we observed the highest sugar production in most of our study sites due to the flowering peak of mass-flowering dandelions (*Taraxacum* agg.) and oilseed rape (*Brassica napus*) that constituted the main nectar producers. Previous studies have shown a positive influence of these crops on bees [32,63,64,65]. Although dandelions were present in a wide range of landscape elements, they were most abundant in intensive grasslands; that offered high nectar sugar production in May, due to their high cover in our study landscapes. In addition, road verges constituted a non-negligible nectar production, due to their diverse and abundant floral assemblage [31,66].

Several *Asteraceae* species (*Taraxacum* agg., *Centaurea jacea*, *Cirsium palustre*, *Cyanus segetum*, *Achillea millefolium*, *Matricaria recutita*) were particularly attractive for insect visitors in our study. Asteraceae are recognized to attract large array of insect visitors due to their copious nectar production [59,67,68,69]. However, *Asteraceae* pollen is not attractive and beneficial for generalist insect visitors due to its low protein content and detrimental secondary metabolites [70,71]. Pollen is the major resource in lipids (including sterols) and proteins (polypeptides and amino acids), but also certainly in secondary metabolites or pesticides that are harmful to pollinating insects [72,73,74,75]. Notably, bees are more selective in their choice of species for pollen resources than for nectar [76].

### 4.2. Extensive Agriculture May Alleviate the June Nectar Resource Gap

Several studies have identified a deficit of resources for pollinators in June [37,77,78]. In our study, we observed a drastic decrease in nectar production in intensive landscapes. Mowing mostly occurred between May and June in intensive grasslands (and spring 2019 was particularly dry and hot). Logically, mowing drastically reduced the availability of the floral resources. June nectar resource gap was far lower in extensive landscapes where extensive grasslands and organic crops covered more than 10% of the landscape surfaces. Extensive grasslands and meadows, in addition to late mowing, provided a wide variety of floral nectar resources for pollinators [59,79,80]. Organic fields provide nectar resources that exert a positive effect on pollinating insects at the landscape level [81]. In our study, cornflower (*Cyanus segetum*) was the main nectar producer. This species is abundant in organic winter wheat fields, where herbicide use is absent [82,83,84]. Cornflowers produce extra-floral nectar before and after flowering, providing resources over a longer period of time for a wide range of species, such as syrphids or pest parasitoids [85].

Our landscape context that include polyculture and cattle rearing is close to the studied landscapes in UK [27]. In such agricultural contexts, extensive practices can therefore be encouraged and propagated in order to compensate for the deficiencies in floral resources, particularly in June. Nevertheless, agricultural practices in these landscapes are a mosaic of crops: cereals, permanent grasslands, fodder crops, and pastures. As a consequence, these landscapes are diversified in their composition and are part of regions with the highest species richness, regarding the spatial distribution of bees in Belgium [86]. The effects of agro-environmental measures on the abundance and diversity of pollinators may be conditioned by the characteristics of the surrounding landscape [29,87,88]. On average, pollinator abundance was higher in intensive grasslands than in extensive grasslands, mostly on *Taraxacum* agg. or *Trifolium repens*, which is an important pollen provider for generalist pollinators, especially for bumblebees and honeybees [47,68].

### 4.3. Nectar Availability only Partly Explained Insect Visitation

Our landscape elements with high sugar nectar production per square meter attract more insect visitors. This is particularly true for species that present both high flower densities and high nectar sugar production, such as *Asteraceae* (*Taraxacum* agg., *Cyanus segetum.*) and *Fabaceae* (*Trifolium* spp., *Cytisus scoparius*) mainly found in open habitats such as grasslands, organic fields or road verges, and *Rosaceae* (*Prunus* spp. *Rubus fruticosus* agg.) in woody elements such as hedgerows and forest edges.

Honeybees generally dominated pollinator observations in *Prunus spinosa* and *Rubus fruticosus* agg. due to their preference for mass-flowering resource patches that sustain colony development, and their evolved social and behavioral adaptations that optimize foraging efficiency for their numbered colonies [89,90]. However, transects were conducted in hedgerows dominated by *P. spinosa*. Only one individual willow tree (*Salix* spp.) was included in our transects and the generally larger size of *Prunus avium* made observations more difficult. As a result, we may have underestimated insect visitation in willows (*Salix* spp.) and *P. avium*.

The overall quantity of nectar seems to explain the number of flower-visiting insects, but this is likely due to the high proportion of honeybees observed in this study. Indeed, the number of observations of visitor insects from other taxa did not seem to be explained by the production of sugar nectar.

Bumblebees, and mainly long-tongued species prefer flowers with relatively deep corolla tubes [49]. This may be one of the reasons why bumblebees have been frequently recorded on *Lamium purpureum*. *L. purpureum* was frequently found in maize stubble in April, where most of the interactions occurred. This species blooms from early spring to late fall and seems to be attractive to wild bees [21,91,92,93]. Because some maize stubbles were not tilled until early May, these landscape elements could provide substantial floral resources for pollinators in early spring, especially in intensive landscapes dominated by arable land. We recommend leaving maize or other cereals stubble in the cultivated fields for as long as possible, in order to provide floral resources thanks to entomophilous weeds.

Nevertheless, pollen resources are also vital for the development and survival of bee larvae [23,74]. Pollen quality, such as the protein content, amino acid profile, protein/lipid ratio, sterol profile [73], or the presence of secondary components [71], influences the floral preferences of pollinators [72]. Notably, bees are more selective in their choice of species for pollen resources than for nectar [76]. In addition to the quality and quantity of floral resources, other factors can influence the floral choices of pollinators. Pollinators can direct their choices according to floral cues (visual, olfactory or tactile) [94,95,96]. The co-evolution between floral species and pollinators may lead pollinators to the specialization on plant traits [97,98]. Thus, the decrease of specific host plants caused by agricultural intensification contributes to the decline of some oligolectic, mainly solitary, wild bees [20,99].

While organic crops had the highest number of flower-visiting insects in June, extensive grasslands had a surprisingly low number of observed pollinators despite their relatively high nectar sugar production per hectare. However, the small number (1106 flower visiting insects) of observations in this study does not allow us to conduct robust conclusions about insect preferences for landscape elements (Table S1). When the transects were carried out in June 2019, the high temperatures may have biased the observations, particularly for bumblebees, as climatic conditions affect foraging behavior [100,101]. *Apis mellifera* accounted for nearly 42% of our insect observations in June during the flowering peak of the extensive grasslands. Honeybee was more often observed at this period in organic crops on *Cyanus segetum*, in hedgerows and forest edges on *Rubus fructicosus* agg. and on *Trifolium repens* in intensive grasslands. However, in extensive grasslands more than half (69%) of the nectar was produced by two species in June (*Cirsium palustre* and *Centaurea jacea*). Still, the number of pollinators observed remained low. This is partly due to the low sampling effort and the reduced number of transects cannot allow us to conclude on the role of landscape elements on pollinator communities. Our results nevertheless reveal the role of these elements in nectar supply. Nectar production seems to explain only partially the abundance of pollinators observed. Therefore, more in-depth studies, especially on the variations in pollen resources in the different regions, would be essential to improve the conservation measures of pollinators. However, there are other elements in agricultural landscapes that can produce resources in spring but were not considered in this study. Trees and forest herbaceous plants can produce abundant amounts of resources [50,59]. Forests are an important element in agricultural landscapes and promote a higher diversity and abundance of pollinators in surrounding landscape [102,103]. As well, gardens can be an important source of floral resources from ornamental species or fruit trees [104], but also a source of nesting site for pollinators [105].

## 5. Conclusions

Extensive grasslands and organic farming practices produce abundant nectar resources in June, during the main colony development, and compensate the decline in nectar resources during this period. However, the contribution of these environments remains relatively moderate early in the season. Hedgerows and forest edges provide most of the resources in early spring, during the colony establishment; intensive grasslands and mass-flowering crops provide large quantities of nectar in May. Floral resources should be assessed for any type of biotope on a landscape scale (thus including woody areas and inhabited areas). Compiling data on the quantities and chemical composition of nectars and pollens of entomophilous species present, as well as their phenology and density, will make it possible to propose differentiated development or management of agricultural areas for the greatest benefit of pollinators and biodiversity as a whole.

## Figures and Tables

**Table 1:** Studied site surfaces, coordinates and proportions of the main landscape elements of the six agricultural landscapes studied. (ext., extensive; int., intensive).

		Extensive Landscapes			Intensive Landscapes		
	Location	Ychippe ext.	Houyet ext.	Wellin ext.	Ychippe int.	Houyet int.	Wellin int.
		5°08′18.61″	4°57′41.62″	5°04′37.94″	5°06′15.64″	4°55′54.19″	5°07′42.38″
		50°14′49.00″	50°10′18.51″	50°04′43.61″	50°14′51.32″	50°09′37.62″	50°04′27.10″
Studied site surface [ha]		195	170	195	251	175	115
Planar landscape elements [%]							
	Crops	44.6	34.6	13.0	44.8	42.8	40.4
	Organic crops	2.6	11.7	1.0	0	0.6	0.9
	Intensive grasslands	39.4	41.1	56.1	53.4	54.3	56.0
	Extensive grasslands	11.0	9.0	25.7	0	0	0
Linear landscape elements [%]							
	Hedgerows	0.4	1.8	2.4	0.5	0.7	0.7
	Forest edges	1.1	1.2	0.8	0.5	0.9	1.4
	Road verges	1.0	0.7	1.0	0.8	0.7	0.6

**Table 2:** Average daily nectar sugar contribution for each landscape element at the landscape scale in a typical 1 ha of the landscape (nectar sugar contribution), and daily nectar production in one hectare of the given landscape element (nectar sugar production). Different superscript letters indicate significant differences among nectar production.

Month	Element Type	Landscape Element	Nectar Sugar Contribution (g ha^−1^)	Nectar Sugar Production (g ha^−1^)
April				
	Linear	Hedgerows	16.0 ± 30.0 ^bc^	1005 ± 1207 ^ab^
		Forest edges	13.0 ± 9.2 ^ab^	1254.0 ± 767 ^a^
		Road verges	0.7 ± 1.5 ^c^	71 ± 159 ^cd^
	Planar	Intensive grasslands	49.0 ± 36.0 ^a^	94 ± 62 ^bc^
		Extensive grasslands	0.3 ± 0.3 ^c^	2.7 ± 4 ^d^
		Intensive crops	2.4 ± 2.2 ^bc^	10 ± 14 ^d^
		Organic crops	0.2 ± 0.3 ^c^	22 ± 37 ^d^
May				
	Linear	Hedgerows	2.2 ± 3.4 ^c^	156 ± 121 ^ab^
		Forest edges	1.2 ± 0.9 ^c^	140 ± 131 ^ab^
		Road verges	2.4 ± 2.0 ^bc^	332 ± 300 ^a^
	Planar	Intensive grasslands	312.0 ± 120.0 ^a^	615 ± 197 ^a^
		Extensive grasslands	20.0 ± 4.0 ^b^	157 ± 86 ^ab^
		Intensive crops	60.0 ± 71.0 ^b^	138 ± 156 ^bc^
		Organic crops	0.1 ± 0.1 ^c^	6 ± 4
June				
	Linear	Hedgerows	1.0 ± 0.9 ^b^	81 ± 57 ^cd^
		Forest edges	1.3 ± 1.3 ^b^	125 ± 90 ^abc^
		Road verges	0.8 ± 0.2 ^b^	112 ± 41 ^bc^
	Planar	Intensive grasslands	66.0 ± 38.0 ^a^	124 ± 53 ^abc^
		Extensive grasslands	212.0 ± 121.0 ^a^	1617 ± 1450 ^ab^
		Intensive crops	9.9 ± 1.9 ^b^	27 ± 55 ^d^
		Organic crops	62.0 ± 79.0 ^a^	2052 ± 1240 ^a^

**Table 3:** Mean daily nectar sugar production at the landscape scale and mean pollinators abundance per site between extensive and intensive landscape.

	Extensive Landscapes	Intensive Landscapes
	Mean ± SD	Mean ± SD
Daily nectar sugar production (g ha^−1^ day^−1^)		
April	105 ± 99	58 ± 34
May	409 ± 113	366 ± 39
June	375 ± 198	82 ± 41
All	296 ± 190	169 ± 152
Pollinator abundance per site		
April	44 ± 18	50 ± 31
May	62 ± 9	31 ± 16
June	93 ± 45	88 ± 31
All	66 ± 32	56 ± 34

## Supplementary Materials

The following are available online at https://www.mdpi.com/2075-4450/11/11/818/s1, Figure S1: Plant species contribution to total nectar supply on each landscape element. Lines show the cumulative contribution of the 10 most productive species in each landscape element. Table S1: Abundance of observed visiting-flower insects in each landscape elements and site per month (ext.: extensive, int.: intensive).
