# Supplementary material for: Contribution of Extensive Farming Practices to the Supply of Floral Resources for Pollinators
Source: Insects. 2020 Nov 20;11(11):818. doi: 10.3390/insects11110818 (PMC7699504; doi:10.3390/insects11110818)
Supplement: Supplementary file 1 [file insects-11-00818-s001.pdf]

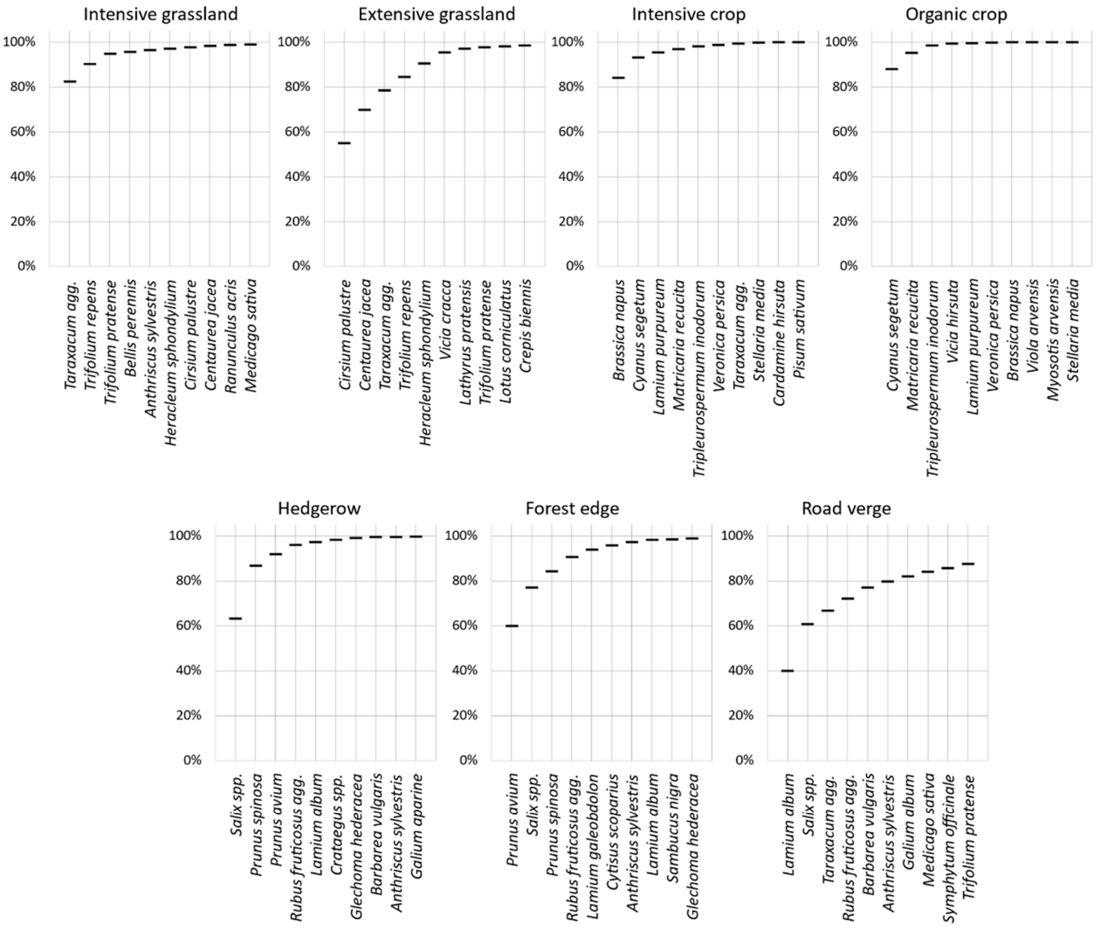

Figure S1. Plant species contribution to total nectar production on each landscape element. Lines indicate the cumulative contribution of the 10 most productive species in each landscape element.

Table S1. Abundance of observed visiting-flower insects in each landscape elements and site per month (ext.: extensive, int.: intensive).

		Number of observed flower-visiting insects																				
		Non-corbiculate bees			*Apis mellifera*			*Bombus* spp.			Other dipterids			Other hymenoptera			Lepidoptera			Syrphids		
Sites	Landscape elements	Apr	May	Jun	Apr	May	Jun	Apr	May	Jun	Apr	May	Jun	Apr	May	Jun	Apr	May	Jun	Apr	May	Jun
**Houyet ext.**	road verge			3		1		1	6				1			3		1	1		1	8
	int. crop		1			7			14													
	ext. crop	2					26	1		8									1			17
	hedgerow				1	2	2	3	1							6				2	1	
	forest edge	1		1		2	3	9	6				1	1			1		3			8
	int. grassland		4	2		1			9						3			1	1		1	
	ext. grassland		5			1		2	2	2		1				2	2		6			
**Houyet int.**	road verge			2			1			3			1									4
	int. crop	7							1		1	2								2	1	
	ext. crop						6						2						1			22
	hedgerow	4			5		2				9			17			1			2		2
	forest edge	6					16		8	2	3			1						2	1	
	int. grassland								4		17									1	1	
**Wellin ext.**	road verge			1				3	3	3												3
	int. crop	1	4		23			19	12											1	4	
	ext. crop			3			7	9	1													1
	hedgerow		7		1	1	25		8	3					2					1		6
	forest edge		2	1			4			2			23		1	14					1	5
	int. grassland		1	3	1	2	1	3	5	3		1							1			1

	ext. grassland		1			1	9		5	3			1					1			6
**Wellin int.**	road verge		2	8		2		2	3			10					2			9	5
	ext. crop		1			2	14	9	2											1	12
	hedgerow		3			2	25	1	3	1					2	2		1		1	6
	forest edge		5	1		1	9	2		1									1		5
	int. grassland						17						3								13
**Ychippe ext**	road verge	1	6		1	16		4	7	3	1		3			1				2	2
	int. crop	15			3			7	9		2			1					2		
	ext. crop			1			14														
	hedgerow	2			1		3						3								2
	forest edge		1		1			1							1						
	int. grassland		4			2			4		3				1						
	ext. grassland			6			1			3								1			
**Ychippe int**	road verge	1	1			1	1	2	3	6			1			1					3
	int. crop	3	1		4	1		16	13											1	
	hedgerow	4			6		5	1			2	4							4	2	5
	forest edge				3		14	5		1											1
	int. grassland			1	2		28	1		4								3	2		3
